# Building and implementation of a common infrastructure for specimen and data storage at an academic medical center

**DOI:** 10.1017/cts.2025.43

**Published:** 2025-03-19

**Authors:** Donna A. Santillan, Laura S. Jacobus, Michael D. Henry, George J. Weiner, Patricia L. Winokur, Boyd M. Knosp, Heath A. Davis

**Affiliations:** 1 Department of Obstetrics & Gynecology, University of Iowa Health Care, Iowa City, IA, USA; 2 Institute for Clinical and Translational Science, University of Iowa, Iowa City, IA, USA; 3 Iowa’s Hawkeye Intellectual and Developmental Disabilities Research Center, The University of Iowa, Iowa City, IA, USA; 4 Roy J. and Lucille A. Carver College of Medicine, University of Iowa Health Care, Iowa City, IA, USA; 5 University of Iowa Holden Comprehensive Cancer Center, Iowa City, IA, USA; 6 Department of Internal Medicine, University of Iowa Health Care, Iowa City, IA, USA

**Keywords:** Laboratory information management, specimens, consent, change management, electronic data warehouse for research

## Abstract

Precision or “Personalized Medicine” and “Big Data” are growing trends in the biomedical research community and highlight an increased focus on access to larger datasets to effectively explore disease processes at the molecular level versus the previously common one-size-fits all approach. This focus necessitated a local transition from independent lab and siloed projects to a single software application utilizing a common ontology to create access to data from multiple repositories. Use of a common system has allowed for increased ease of collaboration and access to quality biospecimens that are extensively annotated with clinical, molecular, and patient associated data. The software needed to function at an enterprise level while continuing to allow investigators the autonomy and security access they desire. To identify a solution, a working group comprised of representation from independent repositories and areas of research focus across departments was established and responsible for review and implementation of an enterprise-wide biospecimen management system. Central to this process was the creation of a unified vocabulary across all repositories, including consensus around source of truth, standardized field definitions, and shared terminology.

## Introduction

Key to translational research are biological specimens [1,2]. While it may appear to be simple, proper documentation of biospecimen collection from research participants, processing, specimen properties, linked clinical data, maintaining data security, storage location, and documentation of informed consent is integral to their usefulness and is not trivial [3–6]. There is little information available regarding successful approaches to harmonize the collection of data elements related to the participants and biological specimens. Having a uniform system for sample documentation even within one academic institution is not easily accomplished and can require a shift in the local culture. Contributing to this difficulty are issues surrounding Institutional Review Board (IRB) approval for what level of clinical information can be retrieved and maintained, a culture of “guarding” access to collections, and unique needs of each research team [7].

While one solution is a core tissue procurement and storage facility, there are usually many departments with collections, each maintaining data locally and with its own domain-specific language and uses. Our academic medical center was no exception [8]. With institutional support, we sought to establish a common solution to store participant and specimen information across the health sciences campus. An ideal solution would lessen the individualized programmatic support required to maintain multiple systems across several projects, reduce costs to investigators, harmonize data, enhance collaboration, prevent redundant collection efforts, and increase the use of stored specimens. Herein, we describe our approach engaging key stakeholders representing multiple departments throughout our university to build a service around a single laboratory information management system (LIMS) using a common data model and implemented across the medical center. This initiative applied an approach, using project management principles and a community-based decision-making approach to 1) Establish a common data model; 2) define comprehensive processes workflows; and 3) establish an enterprise Service Model, defining and established the service to support the tool in a sustainable manner. Our objective is to share the process that we successfully used in order for other institutions to be able to scope the resources needed for similar work and to be able to adapt our approach to fit their specific needs. In the future, this framework could be extended to inter-institutional projects, fostering enhanced collaboration between institutions.

## Methods and materials

Framework: A change leadership approach was taken to progressively build consensus across multi-departmental stakeholder groups including research leadership, researchers, and research support staff [9,10]. This approach iteratively engaged stakeholders to gain consensus on what problems the project was addressing, what solutions to license and implement, and how to implement the solutions in a sustainable manner. Executive sponsorship was established from the leads of large research centers, a project lead and a core team of researchers and support staff was formed. A project lead guided the change leadership process.

Engagement of executive team: The project lead met with each executive committee member individually to establish key outcomes for the project. Through an iterative process, this information was collated, summarized, reflected to the executive steering committee, and used to establish the formal charge and outcomes of the project.

Identifying key stakeholders and teams: A project lead supported the project and provided guidance and structure for implementation with the core team. In addition, the project lead liaised with the executive team to reporting progress. The project lead returned questions or Generating System Priorities: The first task was for each core team member to describe their lab’s current systems and workflows for managing data and specimens. They presented the scope of their collections, including the number and types of specimens, study participants, and associated data. Groups prioritized data elements into “needs,” “would like to have,” and “can sunset,” and explained the strengths and weaknesses of their current systems.

Identification of common data elements: To harmonize data and create common data elements, the project lead led the core team through several sessions to establish consensus. Lists of groups’ data elements were prepared, and the core team aligned the data elements and definitions to identify overlap. For each variable that was collected by more than one group, a discussion was held to reach consensus on the format for future storage.

In the consensus-building process, it was essential to represent data elements sustainably and usefully for future research teams. This involved reviewing the literature for common terminology. A data dictionary listing these common data elements was created. Unique data elements were discussed to determine if they could benefit other groups or be captured differently. Groups could choose to maintain unique variables if necessary. The team also considered potential future data elements and used “Process Flow Maps and Diagrams” to decide how and when data would be entered for each specimen type.

Review of informed consent: The core team provided 35 examples of informed consents used at the institution and outside of the institution for specimen collection and biorepositories. A subset of the core team reviewed the breadth and scope of these to develop and implement an ontology and data-model to represent what use a specific specimen was collected under in the system.

Generating system requirements: In addition to defining data elements, system requirements were generated by core team members and their stakeholders. Each domain generated a list based on current and future envisioned workflows. Information technology provided recommendations and requirements around systems, security, redundancy, and institutional policies and practices. Compliance provided legal and policy guidance. Requirements were categorized according to MoSCoW method and “must have,” “should have,” “could have,” “will not have,” then prioritized ordinally by the core team [11].

Request for proposals: Following institutional practices for large purchases, a request for proposals (RFP) was generated. The RFP included system requirements for data security and the needs identified by the core team including: specimen lifecycle tracking, flexible query and report management capability, storage visualization, and ease of moving specimens between facilities, tracking of the consent version a specimen was collected under to understand the scope of use, integrated functionality with electronic health records (EHR) or other systems, and ease of importing large amounts of data to allow for onboarding of new labs and sunsetting of legacy systems. Detailed requirements that were identified by the university, hospital system, and future users are provided in the Appendix, System Requirements and Functional Requirements.

Selecting the LIMS: Following receipt of completed proposals from vendors, the core team reviewed responses and rated them based on a common review criterion. Proposals with the highest scores were invited to present their product to the group. RFPs were reviewed and selected for web/in-person demonstrations. After the initial review, the team then chose to perform a more in-depth review of 4 products. The core team hosted web calls or in-person demonstrations with potential vendors. The core team scored systems based on the demonstrations and functionality of each LIMS. The four top choices were asked to provide test instances for hands-on evaluation by the core team. Each member of the core team ranked the software programs. The top three options were presented to the executive team by the project lead with the core team present. A list of pros, cons, and return on investment was included in the presentation.

Defining the service: To complement the implementation of an enterprise LIMS, the core team identified a need for a local expert in the software to train the users, liaise with the vendor, test and implement updates, transform and upload previously collected data, design common templates and individualized forms, act as an honest broker for providing data, and maintain the centralization of the system. It was determined that this role would be held by a full-time Service Manager, tasked with support of the new “UI BioShare” service. The core team considered the nuances of what the service provides and how it should be supported. Specific terms negotiated included acceptable service costs both per deliverable and as a departmental support mechanism funded through grants, desired services, what is actionable by users themselves versus what would benefit from service level guidance and support, and finally, how to market the service for high visibility and ease of service request activities both internal and external to the institution. Critical to this role was that, in addition to technical skills, domain expertise, the individual could manage and navigate the business relationships between the cores, potential users and leadership moving forward. Members of the core team were involved in developing the position description and interviewing candidates.

Implementation: The core team participated in several working group meetings to discuss, test, and finalize acceptable workflows. Based on the workflows and the requirements of the system, common fields and forms were defined that would be universally implemented at University of Iowa Health Care. After common elements were defined, new requests for changes to the common forms by any user continue to require approval by members of the working group before implementation by the Service Coordinator.

**Figure 1.**
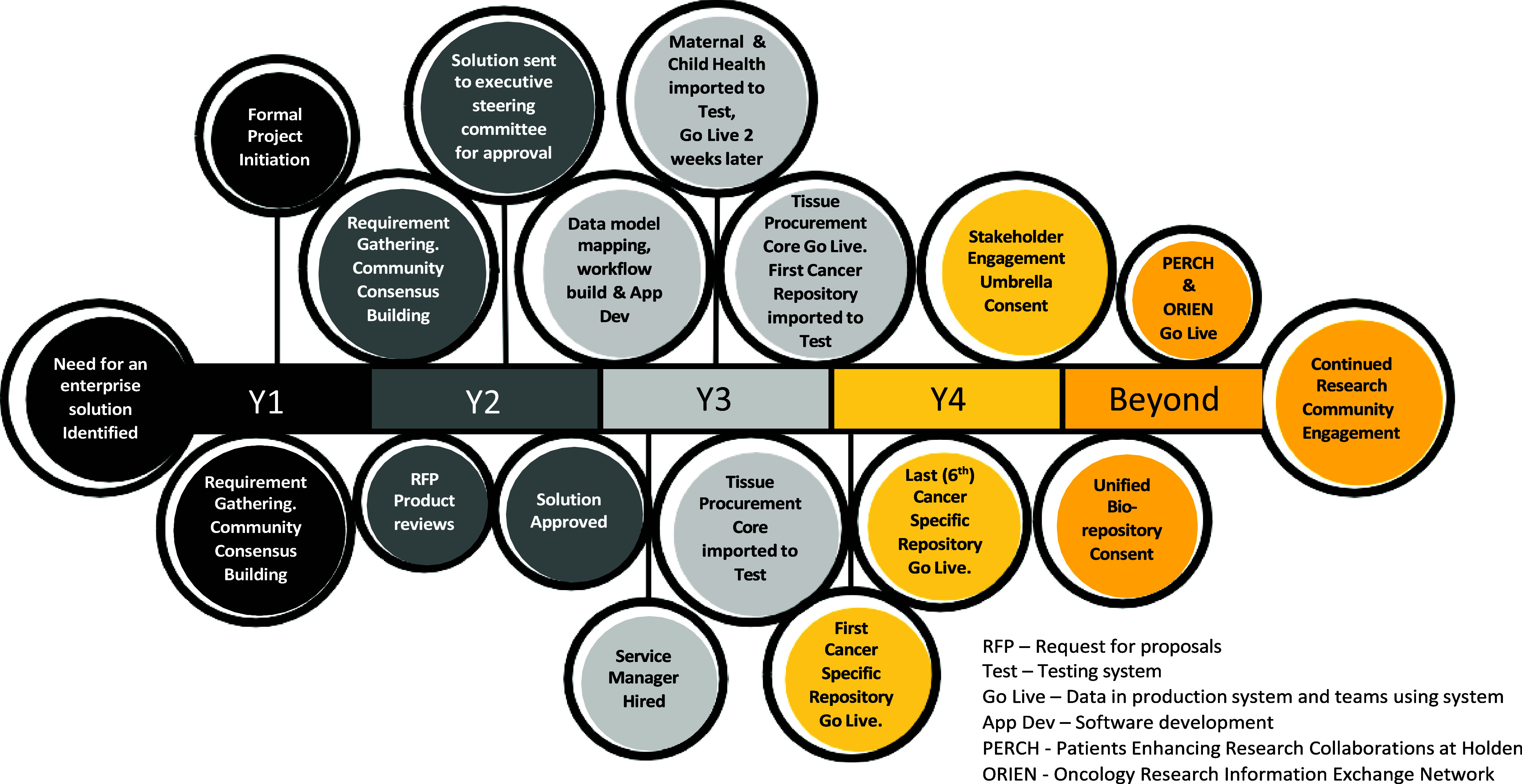
Project timeline. This was a multi-year project from idea conception through implementation.

The core team designed common forms within the architectural framework of the LIMS while also allowing individual groups the flexibility to design forms unique to their content needs. Using an iterative and incremental software development (Agile) approach [12], the Biomedical Informatics team worked with individual groups to extract data from current systems, transform data to match the new data dictionary, clean and validate data, upload to the Test system, validate data, and then upload to the production instance of the new system. Double data entry into old systems and the new system was performed for one month by each group after their “go-live.” At that time, data veracity was confirmed, and double data entry was discontinued. Figure 1 summarizes the project timeline.

## Results

In a blind vote, the working group unanimously selected the same LIMS. This choice was then presented to the Executive Committee with all members of the working group present to address questions. The purchasing department then negotiated contract terms. Pragmatic features of importance included in the negotiation included the number of licenses, support time for development and transition, ability to use application programming interface to extract information from the EHR, and the capability to extract all data should the company cease operations.

Throughout the phased go-live, the service team worked with three of the largest user groups, the Cancer Center’s Molecular Epidemiologic Resource groups, Obstetrics and Gynecology’s Women’s Health Tissue Repository and Pathology’s Tissue Procurement Core to migrate 21,000 enrolled research participants, and 150,000 aliquot or vial level specimens. In addition, 62 facilities, 30 storage units comprised of variable temperature level units including 14–80° Celsius Freezers, 13–140° Celsius or Liquid Nitrogen Vapor tanks and chest freezers, and 3 ambient slide storage chests and boxes were migrated. Since the initial go-live efforts, > 114,000 research subjects and 332,000 aliquot or vial level specimens, and 560 studies have been added to the system (Table 1). Only 25 domain-specific forms have been created. There are currently 18 research groups with > 380 users who have been active in UI BioShare. Eight disparate systems used to catalogue research specimens and related patient data have been sunset, saving resources needed to maintain multiple systems and allowing expertise to grow across the community.

**Table 1.** UI bioshare metrics

Study	Subject	Specimen	
Study ID	Medical Record #	Study and Donor ID	Collection Container
EHR Study ID	Name	Specimen Type	Disease Status at Collection
IRB Number	Date of Birth	Specimen Site/ Derivative	Treatment Time Point
IRB Dates	Data of Death	Specimen Status	Line of Therapy
Approval Status	Mortality Status	Date of Collection	Pathological Diagnosis
Study Roles/Rights	Sex	Date Processed	ICD10 /SNOMED Code
Study Team	Ethnicity	Date Preserved	Reserved Status
Consent Parameters	Race	At Facility	Surgical Details
Study Status	Street Address	Storage Location	Quality Assurance
	City, State, Zip	Thaw Count	Extraction Method
	Phone Number(s)	Initial Quantity	Molecular Results
	Email	Sample State	Current vol, Concentration or Mass
	Consent Status	Preservation Media	
	Date of Status	Processed By	
	Consent Date	Problem / Problem Notes	
	Consent Version	Collection Mode	

The above information was migrated for each study, subject, and specimen. These details are also maintained as new studies, subjects, and specimens are added. ID = Identification; IRB = Institutional Review Board; Vol = volume.

**Figure 2.**
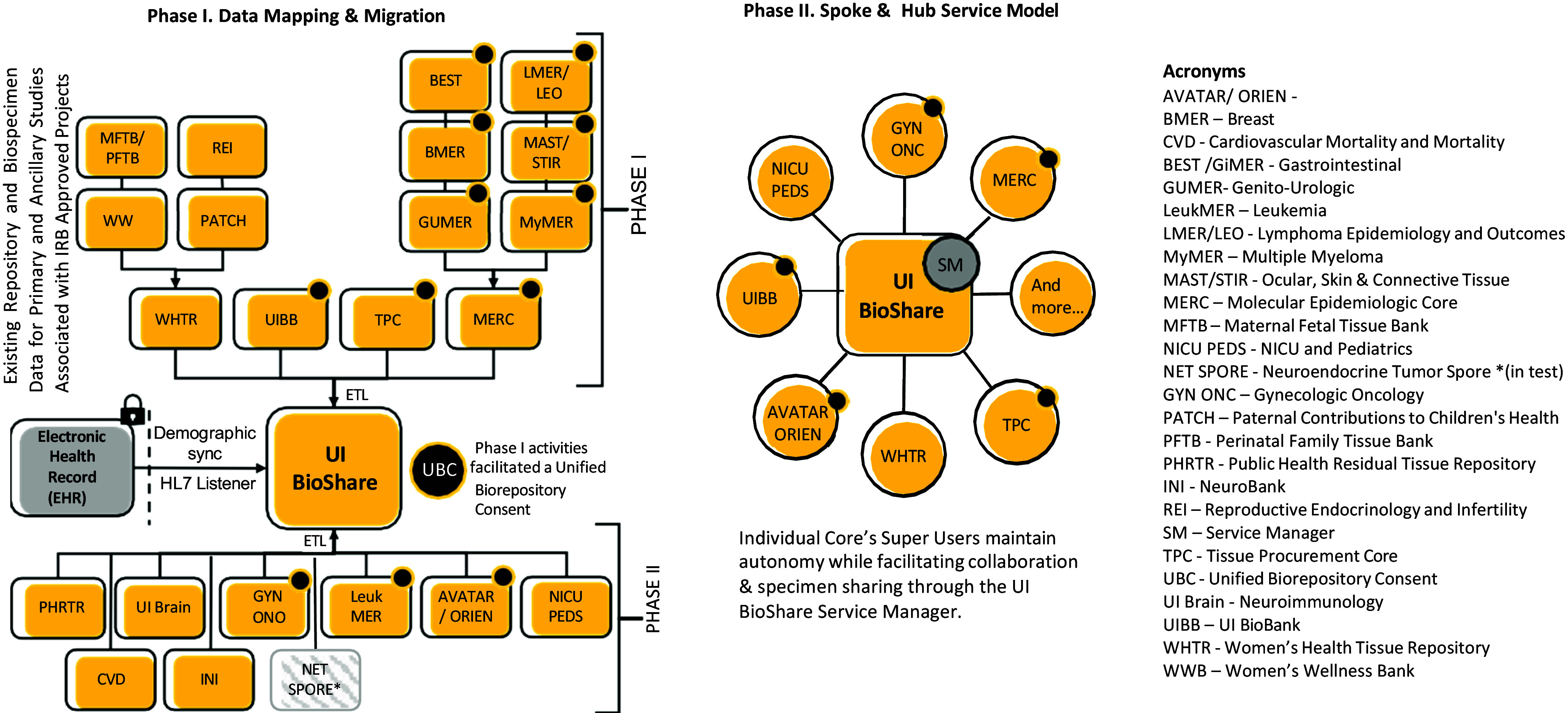
Project design. program implementation occurred in two phases. In phase I, data was mapped and migrated. In phase II, the service model was matured and new users were added.

**Figure 3.**
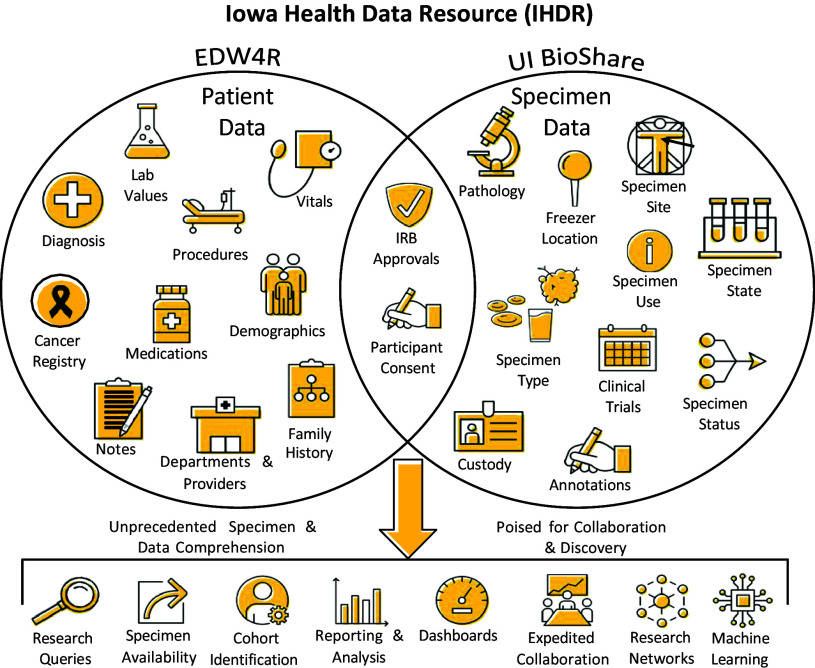
UI bioshare concept. The UI bioshare service has resulted in many benefits to the university community. By connecting with information in our electronic data warehouse for research, we have reduced duplication of efforts and improved our ability for collaboration and discovery. EDW4R = Enterprise Data Warehouse for Research; IRB = institutional review board.

By transitioning to an enterprise level system, we have tracked specimens for their lifecycle. Documentation of 33,500 specimens transferred to investigators and facilities for research have been documented. We have helped to establish long-term collaborations between the members of the core team by referring faculty to other research groups with similar research interests. As this project was a large undertaking, it was divided into two phases (Figure 2). In the first phase, the service was defined and implemented. In the second phase, we sought to expand the capabilities of the service. Some of these expansion efforts depended on upgrades that were forthcoming from the company (bulk operations, family linkage and pedigree, invoice operations, API functionality). Other “wants” by the team that were not required prior to go-live were deferred to Phase II until all initial participants had been onboarded. Non-urgent projects that were delayed to Phase II involved continuing study migration of additional repositories, enhancing and maturing the service, continuing work with institutional research offices and security custodians of the electronic medical to develop ways to work with the Iowa Health Data Resource (IHDR) and the Enterprise Data Warehouse for Research (EDW4R), to decrease the need for data entry by research teams of data that is already available in the medical record such as patient clinical data and test results (Figure 3). Phase II also included the initiation of a Scientific Community Outreach service introducing UI BioShare to various departments, research groups and colleges. To mature the service and tool, other phase II activities included regular review and maintenance of “Minimal Footprint” for all users to adhere to regarding data entry, development of consent template with Institutional IRB for biorepository use of UI BioShare standardizing repository specimen and data language and the maturation of change management process used for the UI BioShare service and toolset.

## Discussion

We utilized a community-based decision-making approach to designing and implementing a central biospecimen information system that has been widely adopted at UI. Key factors to the success of the project included leadership sponsorship, engagement of key stakeholders, and a project lead who iteratively led the core team through consensus decision making tasks throughout the project. This approach has become a model for other successful initiatives at UI and can be adapted to fit local needs at other sites.

From our early meetings with potential users, it was clear that cost was one of the main factors in whether individual groups would consider using the UI BioShare program. Several labs were using spreadsheet software or database management systems that are included in suites of software installed on campus computers because they were free. There was a strong concern that if a lab lost funding that they would lose access to their participant and specimen information. Labs were also opposed to purchasing access to new software if they already had a software system in place and did not want a large expense to migrate information to a new system. As a result, it was decided to subsidize service costs to provide it to any UI groups free of charge. As a free service, new groups were more likely to utilize this system. Current biobanks were also provided no cost assistance to transfer their current data into the new system. The current biobank collection data were reviewed for accuracy after fields were transformed and after a test small-batch upload from a small batch of samples was performed. After this confirmation, the rest of the information was migrated. Therefore, other groups seeking to implement a similar service should strongly consider its startup funding costs and sustainability.

There were several unintended, but additional benefits from this project. By holding weekly meetings of the core team, this group developed a familiarity with the specimens and cohorts available on campus. The core team members became a tremendous resource for their departments for knowing who to contact and preventing duplication of collection efforts. In addition, the core team members took advantage of learning about the workflows of the other groups and optimized their own specimen and data collection. There is now more collaboration across departments and research teams for patient enrollment and collection of research specimens. Similarly, maintaining an enterprise level LIMS increased visibility of repositories and research teams that exist across campus. This information is extremely helpful when applying for institutional or center grants. We have leveraged this approach combining Community Based Decision Making, Project Management Fundamentals and having a diverse set of subject matter experts across science and technology in several other successful projects [13,14].

**Figure 4.**
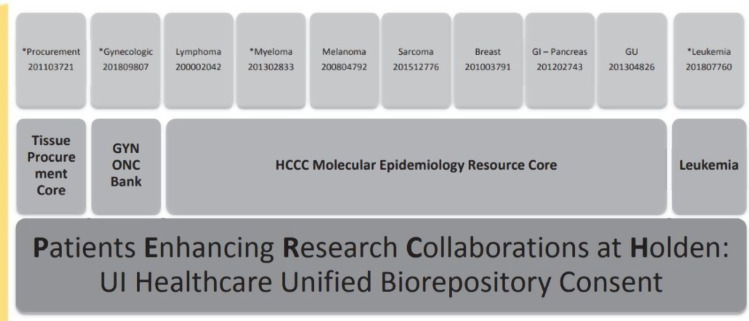
Unification of collections at the Holden Comprehensive Cancer Center. By providing a uniform consent and storage process for all cancer-related specimens, the University of Iowa can easily participate in new collaborative opportunities. GI = gastrointestinal; GU = genitourinary; GYN ONC = gynecologic oncology; HCCC = Holden Comprehensive Care Center.

The existence of an enterprise-level LIMS allowed for efficient and expedited collaboration with external consortium groups. Having the cancer-specific biorepositories catalogued in UI BioShare put our Cancer Center ahead of the curve when joining a partnership of Academic Health Centers focused on Precision Medicine research. While many of the other centers built their programs from scratch, taking years to establish workflows and supportive infrastructure, UI was able to build on existing infrastructure and merge the information across the cancer-specific biorepositories into a single project and contribute within months of signing the agreements (Figure 4). In the first three years of participation, we were able to provide 250 matched cases that met the project-specific stage and diagnosis eligibility. This would have been tenuous without a centralized resource for cataloguing research specimens and would have taken many years to grow prospectively. In addition, preparation for this collaboration included creation of a comprehensive biorepository consent that required the consolidation of eight disease-specific biorepositories; the infrastructure within UI BioShare allowed for accurate capture of participant consent status and documentation across studies simplifying review of permissions across consent versions and studies. This information provided the data and infrastructure required by the Institutional Review Board to merge the legacy disease-specific biorepositories under a single Cancer unified biorepository consent resource, “Patient Enhancing Research Collaborations at Holden.” Close to 10,000 participants have been enrolled since its inception in November 2017. Before this project began, the management of biospecimens and biobanks was handled by separate research laboratories or groups within departments. These entities had varied levels of technical expertise and varying control and backup processes. However, with the development and implementation of a unified system for managing biospecimens across health care, the management has been centralized. This centralization has been facilitated by secure servers, firewalls, roles, groups, and centralized credential management, simplifying the secure management of the system, and ensuring the regular execution of systematic backups.

Focused engagement of the core team facilitated decision making regarding common workflows and increased community-based decision making across teams. These stakeholders were familiar with the diverse processes of each group member and the possibilities based on the structure of the software. If an open forum process had been used, it would have yielded results that were less practical to implement. Further, the time that the core team spent together fostered a collegial atmosphere and a willingness to ask questions, make suggestions, and challenge ideas. Therefore, despite the investment of time by each core team member, they found it to be a beneficial experience.

The time dedicated to the meetings and implementing this service also drove the core team’s desire for a successful implementation of UI BioShare. Individual group members worked to clean their data to be ready for each phase of implementation. Each independent unit was implemented separately to allow the Informatics team to learn from each process. The core team continues to serve as champions of the service and direct new lab groups to the UI BioShare Service Manager for consultation and eventual “go-live” within the system.

The Service Manager acts as an honest broker. If a researcher has a request for specimens, the Service Manager can retrieve information about who may have the needed specimens and direct the researcher to the correct contact(s). Collaboration or specimen distribution is then at the discretion of the individual group and domain-specific oversight committees are responsible for scientific and resource evaluation to grant project or distribution approvals.

Because the software system costs would be prohibitive for any one group, having many users at the medical center justifies the investment costs. This service has benefitted the medical center by enabling researchers to identify new collaborations and for the inclusion of powerful information regarding specimens and participants for grant submissions. Furthermore, a single system has reduced the number of LIMS that need to be maintained by the biomedical informatics group and allowed the group to focus its resources to maintaining and expanding the UI BioShare service.

Acceptance of this service has been highly successful. Groups adopted the infrastructure because they retained control over their specimens and data, without centralizing storage. The core team included language in their IRB protocols to permit specimen and data sharing between groups, reflecting a significant cultural shift at the University. This initiative was conceived and implemented by the core team independently, without direction from the Executive Team. The Tissue Procurement Core continues to be available as a fee-for-service core to consent, collect, and distribute tissues. While labs are not required to utilize core resources, in close collaboration with the Department of Pathology, the Tissue Procurement Core does act as the sole handler of tissues being distributed from Gross Pathology. This process reduces confusion and allows Pathology to complete their clinical work and gives researchers one point of contact more easily. We have created a spoke and hub model for specimen collection and storage that allows each group to maintain its independence but be connected to the UI BioShare system.

The system’s implementation was smooth overall but had some weaknesses that can be optimized at other institutions wishing to implement a similar service. 1) Inconsistencies arose from multiple people transforming data for uploading into UI BioShare. 2) Convincing new researchers to store data in UI BioShare is challenging, as they often wait until they have a large collection, adding extra work for data transformation and loading. 3) Not all researchers are required to use UI BioShare, so some collections may not be captured. To reduce the occurrence of these challenges, 1) the lead can write clear guidelines for data transformation and be the point person for any questions related to uploading data, 2) advertise the service broadly to existing researchers and inform new research hires of the service availability, and 3) institutions can reduce or sunset support for competing, similar services.

Despite the significant resources needed for design and implementation, the University has seen clear benefits. A similar implementation plan would be valuable for other institutions to facilitate specimen and data exchange.

## Supporting information

Santillan et al. supplementary material Santillan et al. supplementary material

## Supplementary material

The supplementary material for this article can be found at https://doi.org/10.1017/cts.2025.43.
