# Supplementary material for: Building and implementation of a common infrastructure for specimen and data storage at an academic medical center
Source: J Clin Transl Sci. 2025 Mar 19;9(1):e76. doi: 10.1017/cts.2025.43 (PMC12083205; doi:10.1017/cts.2025.43)
Supplement: Santillan et al. supplementary material [file S2059866125000433sup001.docx]

Appendix

System Requirements:

1. Study creation.
   1. Ability to define a study/project with all relevant details (including IRB details)
   2. Ability to share the study templates (basic annotations) across studies.
2. EHR API Interface to:
   1. Receive alerts from the electronic health record (EHR) to collect specimens.
   2. Ability to import demographic information.
   3. receive and store the de-identified copy of surgical path report in biobank (after a surgical path report is completed in EHR).
   4. Once the patient is consented ability to import the additional diagnosis, labs etc. information from EHR.
3. Managing information retrieved from EHR.
   1. Saving information retrieved from EHR into biobank.
   2. Setup markers to indicate patients need to be approached for consent.
      1. Test case 1. If the patient already exists in the biobank.
      2. Test case 2: Patient does not exist in the biobank.
      3. Test case 3: Handling data mismatch in already existing patient in the biobank.
4. Consent Management
   1. Managing consent and decline date.
      1. Test case 1: What if a patient already exists in a system who has declined for the same study, how does system handle this scenario?
   2. Test Case 2: What if a patient already exists in the system but has declined for a different study?
   3. Test Case 3: What if patient cannot / could not be approached for consent?
   4. Ability to scan and store consent documents in the Biobank repository.
   5. Ability to track when patient declined last time and determining when a patient can be re-approached.
5. Question: How do we determine eligibility of patient (is this a manual process)? Do we need a mechanism to automate this?
6. Data Sharing / Automation
   1. Ability to share the list of patient medical record numbers (MRN) with other groups (like Pathology) to collect specimens (whenever available)
   2. Ability to share the identified and de-identified version of patient data.
7. Specimen Management:
   1. What if specimens could not be collected for some reason?
   2. System should be able to track when the specimen was collected, received, not collected, damaged and along with the associated cause/reason.
   3. The system should be able to track features associated with each specimen collected (weight, dimensions, gross description, number of pieces per mold or foil, number of molds or foils, disease status of each mold or foil, ischemic times, etc.)
   4. Recording and tracking how specimen was stored.
   5. Defining custom storage locations and being able to store specimens in defined hierarchies (Freezer, Rack, box etc.)
   6. System to allow creation of aliquots and derivatives for distinct types of specimens and each aliquot should inherit features of the parent specimen.
   7. The system should provide functionality to reserve the storage space and available specimens.
8. Specimen sharing
   1. Users should be able to query and request for specimens (providing rational for specimen request, related PI (Principal Investigator) and requested volume, required by etc.).
   2. If the specimen is available in the biobank, the system should create a workflow and track the request till it gets approved, and specimen is shared.
   3. System should generate required emails to notify its approval/reservation/denial.
   4. System should also adjust the inventory count and release the storage space after specimen is shared.
      1. Test case 1: checking storage space availability after specimen is shared,
      2. Test case 2: checking storage space availability to a particular group who has reserved that space in the freezer.
   5. The system should provide functionality to capture shipment and local specimen sharing.
9. Reporting
   1. Ability to query and check availability of specimens.
   2. Reports to consolidate the billing should be available.
   3. Report to check available / occupied storage space.
   4. Reports to extract study patient data
   5. Reports to track overall biobank reports (for ICTS / senior management to evaluate its effectiveness)
   6. Custom reports to check the available specimens and volumes in a study.
   7. Printing bar codes
10. Invoices and Billing
    1. Required functionality to manage billing should be available.
11. Ability to export data out of biobank for Longitudinal analysis.
    1. Being able to track the consented / declined patients separately. Also ensuring that system stores minimum data of patients who have declined.
    2. Ability to handle a clinical trial where multiple specimens are collected vs. studies that collect one-time specimens along with custom annotations attached with each specimen collection.
    3. Ability to track these types of specimens with different measurement units and storage requirements etc.
12. Data management (including Migration and Integration)
    1. Flexibility to migrate data from existing projects and applications.
    2. Managing API’s from EHR/ other applications
    3. Managing bulk data import / export
13. DNA Processing
    1. After the above procedures are done to register patient enrollment and log the initial blood or saliva specimen, lab personnel take over and begin a new workflow.
    2. System should allow flexibility and special workflow management for DNA specimens (as below)
    3. Blood is spun down and plasma aliquoted into 2 mL tubes for storage, barcoded, and storage location noted in database.
    4. Depending on initial blood volume, 2 mL or 250 ul are aliquoted for DNA extraction, in batches of 8 specimens / extraction. Scanning specimens to create an extraction batch would be useful.
    5. Depending on remaining blood volume, blood spots on filter paper are created, barcoded, and stored with storage location noted in database.
    6. Depending on remaining blood volume, 2 mL aliquots of blood are created, barcoded, and stored with location noted in database.
    7. Entire saliva specimen is used for DNA extraction.
    8. Once DNA is generated, it is stored in 2 aliquots (-80 stock and working stock)
    9. Date processed, tech doing processing, volume of each aliquot and storage location of each aliquot tracked as well as initial volume of specimen, DNA concentration and 260 and 280 OD readings or nano drop readings imported.
    10. XY genotyping for QA purposes run and entered the database and compared with the participant gender to ensure a match.
14. Barcoding features should be available.

Functional Requirements:

- 1 SPECIMEN MANAGEMENT
  - 1.1 SPECIMEN TYPES
    - 1.1.1 Ability for TPC/UI Biobank to define specimen types within the biobank system that would be used by all projects.
    - 1.1.2 Ability for TPC/UI Biobank developers to create additional specimen types as needed.
    - 1.1.3 Ability to flag a specimen type which is not part of the institutional terminology as a sub-type or missing type in that terminology.
    - 1.1.4 Ability to maintain genealogy information on specimens.
    - 1.1.5 Ability to maintain genealogy across specimen types.
  - 1.2 SPECIMEN CONTAINERS / STORAGE UNITS
    - 1.2.1 Ability for TPC/UI Biobank to define standard Container types and Storage Unit types based on institutional terminology or ontology.
    - 1.2.2 Ability to support Containers for both liquid and solid specimen types and Storage Units used at Univ of Iowa
    - 1.2.3 Ability to associate attributes to Containers and Storage Units
    - 1.2.3.1 Ability to specify Additives/preservatives of a Container.
    - 1.2.3.2 Ability to define the dimensions of a Container and a Storage Unit, using up to 3 dimensions (Length, Width, Height, Volume)
    - 1.2.3.3 Ability to define capacity, including volume and mass it can contain.
    - 1.2.4 Ability to specify Container and Storage Unit hierarchy: i.e.: Ability to specify what specimen type can be held by a container; Ability to specify what Container can be held by a Storage Unit; Ability to specify what type of Storage Units(s) can be held by other Storage Units
  - 1.3 SPECIMEN LABELS
    - 1.3.1 Ability to scan system generated barcodes and identify the specimen within the container.
    - 1.3.2 Ability to scan externally generated barcodes of imported specimens and identify the specimen within the container.
    - 1.3.3 Ability to provide barcode label printing capabilities or seamless integration with barcode printing tools.
    - 1.3.4 Ability to support Linear and 2D barcode formats and RFID’s.
    - 1.3.5 Support standard barcode printers
    - 1.3.5.1 Ability to interface with standard label printing software.
    - 1.3.6 Ability to ensure unique IDs across the system.
    - 1.3.7 System provides label text that is human readable and contains scannable barcode; scalable to label size.
    - 1.3.7.1 Ability to specify which printer to use.
    - 1.3.8 Ability to print to local or networked label printers.
    - 1.3.9 Ability to track multiple external UIDs and barcode IDs for each specimen.
    - 1.3.9.1 Ability to use legacy or 3rd party barcode IDs without re-barcoding the tubes.
    - 1.3.10 Ability to store and print UTF-8 characters on labels.
    - 1.3.11 Ability to support labels in use at each biobank and the TPC/UI Biobank
  - 1.4 SPECIMEN LOCATION
    - 1.4.0.1 Ability for system to automatically assign storage locations based on specimen type to next available appropriate location or to manually choose locations from a generated set of options.
    - 1.4.0.2 Ability to display storage locations graphically and for the user to select and query locations from the graphical display.
    - 1.4.1 Ability to support a location hierarchy for specimen storage.
    - 1.4.1.1 Ability to track and locate a position within a container (2D or 3D matrix) and to identify what is stored in the location.
    - 1.4.2 Ability for the system to track and maintain a location history for each specimen and container, even if specimen is removed or depleted.
    - 1.4.2.1 Ability for privileged users to view specimen location histories and incorporate these histories in queries for reports.
    - 1.4.2.2 Ability for privileged users to view who removed or inserted a specimen and the timestamp.
    - 1.4.3 Ability for automatic workflow features that update status of locations when removed from inventory by lab staff (e.g., destroyed or shipped)
    - 1.4.4 Ability to identify where available storage locations exist at both the box, rack, and freezer levels.
    - 1.4.4.1 Ability to request available storage locations for a user specified number of containers, either contiguous or not
    - 1.4.4.2 Ability to prioritize the display of available storage locations based on protocol to store containers from the same protocol together.
    - 1.4.5 Ability to assign or change locations of multiple items at the same time, including specimens into a box, or boxes into a rack, etc.
    - 1.4.6 Ability to quickly reassign the location of a group of specimens.
    - 1.4.7 Ability to assign locations manually by overriding automatic system assignment of location (e.g., filling available locations)
  - 1.5 SPECIMEN ATTRIBUTES & ANNOTATIONS
    - 1.5.1 System has a mechanism to collect pre-defined and institutionally defined specimen attributes, annotations, and business rules required to support workflows.
    - 1.5.2 Ability for the TPC/UI Biobank administrators to associate attributes to specimens by bank or project.
    - 1.5.3 Ability to configure ad hoc attributes that are flagged as not part of the TPC/UI Biobank terminology.
    - 1.5.4 Ability to maintain a map between local terminology (of a bank or project) and institutional terminology.
    - 1.5.4.1 Ability for the TPC/UI Biobank to define a required set of rules, attributes, and specimen data elements or specimen attributes for all protocols, while allowing individual banks to add to those variables or attributes.
    - 1.5.4.2 Allows administrators to specify attributes in addition to TPC/UI Biobank required attributes for each specimen type for a bank or project.
    - 1.5.4.3 Ability for events to be triggered if attribute values deviate from a defined set of values or range of values.
    - 1.5.4.4 Ability to support branched logic or alternate forms defined by specimen type-specific data.
    - 1.5.5 Ability to create ad-hoc attributes isolated to a specific bank, project, or workflow and then to associate data with those attributes.
    - 1.5.6 Ability to register multiple specimens to a given participant, project, tissue without data re-entry for common/hierarchical data elements.
    - 1.5.7 Ability to automatically update specimen quantity when aliquot or derivative is made.
    - 1.5.8 System allows the ability to configure alerts based on defined attributes that would limit a specimen's availability or quality.
  - 1.6 SPECIMEN RECEIPT & INHERITANCE
    - 1.6.1 Ability to specify specimen attributes specific to shipping and receiving.
    - 1.6.2 Ability to import project, specimen collection, and timepoint information.
    - 1.6.3 Ability for derived aliquots and specimens to inherit attributes from parent(s)
    - 1.6.3.1 Ability to inherit participant attributes associated with the parent specimen.
    - 1.6.3.2 Ability for specified specimen attributes to be inherited from the parent specimen.
    - 1.6.3.3 Ability for attributes on a group of specimens to be updated with a single change.
    - 1.6.3.3.1 Ability for children of a parent specimen to be treated as a group (as above) and attributes updated or revised as a group.
    - 1.6.3.4 Ability for the TPC/UI biobank to specify which attributes of the parent are or are not inherited by child specimens, aliquots, or derivatives.
    - 1.6.4 Ability to attach free text comment fields to specimens, events, workflows, etc.
    - 1.6.5 Ability to search or process free text comment fields using Natural Language Processing (NLP) tools.
    - 1.6.6 Ability to set standard volume setting for container(s) to accommodate volume preferences at project level.
    - 1.6.6.1 Ability to assign a child or parent relationship after both specimens are independently registered.
  - 1.7 SPECIMEN ACCESSIONING & TRACKING
    - 1.7.1 Ability to track specimen information for all aliquots and derivatives created in the TPC/UI Biobank and potentially by other core facilities or research labs within the greater institution, including those not currently stored within the TPC/UI Biobank.
    - 1.7.2 Ability to maintain participant, specimen and aliquot tracking information as a specimen moves between biobanks and projects.
    - 1.7.3 Ability to reassign ownership of specimens or to move specimens from one project to another.
    - 1.7.4 System can generate/assign a unique accession number to each specimen.
  - 1.8 SPECIMEN SHIPPING
    - 1.8.1 Ability to track information about the shipped specimen.
    - 1.8.2 Ability to build and add specimens to a shipping container using a template, based on predefined containers.
    - 1.8.3 Ability to build a report or shipping manifest to accompany the shipping container.
    - 1.8.4 Ability to record Material Transfer Shipment (MTS) data if part of a transfer or shipment.
  - 1.9 SPECIMEN REACCESSIONING
    - 1.9.1 Ability to store attributes on accessioned and re-accessioned specimens.
    - 1.9.2 Ability to programmatically inherit specimen history prior to re-accessioning.
    - 1.9.3 Ability to support the representation of specimen inventories from biobanks external to the TPC/UI Biobank
    - 1.9.3.1 Ability to import a read only copy of data through either a ‘data dump’ or direct API from an affiliated biobank and view all specimen and project data within the TPC/UI Biobank.
    - 1.9.3.2 Ability to configure rules for updates to data from the 3rd party biobanks.
    - 1.9.4 Ability to import tracking information from an external system about specific aliquots managed by the external system, and store and report tracking information about those aliquots.
    - 1.9.5 Ability to batch import specimen data.
    - 1.9.5.1 Ability to batch import specimen data including an indeterminate number of recursively linked specimens that may exist in a parent-child hierarchy.
  - 1.10 SPECIMEN DISTRIBUTION & DESTRUCTION
    - 1.10.1 Ability to track attributes associated with distribution and destruction.
    - 1.10.2 Ability to define the maximum retention time of a specimen or a retention schedule.
    - 1.10.2.1 Ability for the system to execute a workflow based on the expiration of a specimen's maximum retention time or retention schedule.
- 2 STUDY & PROTOCOL MANAGEMENT
  - 2.1 Ability to define and track a protocol in the system.
  - 2.2 Ability to link zero or one or many IRB studies to a protocol.
  - 2.3 Ability to restrict each specimen to be governed by only one protocol at a time.
  - 2.4 Ability to import projects from another informatics system or bank.
  - 2.5 Ability to delegate some level of administrative control to a local protocol delegate.
  - 2.6 Ability to define TPC/UI Biobank level attributes for a protocol.
  - 2.7 Ability to define protocol-specific attributes of a protocol.
  - 2.8 Ability to view and export specimens grouped together by protocol attributes.
  - 2.9 Ability to use protocol templates to apply project attributes to a protocol.
  - 2.9.1 Ability to copy an existing protocol structure into a new project without copying the subjects or specimens.
  - 2.9.2 Ability to modify protocol templates by adding or removing protocol attributes.
  - 2.9.3 Ability to inherit protocol attributes and content for subprojects or a protocol.
  - 2.10 Ability to define biobank user security rules based on individual workflow and procedures.
  - 2.11 INFORMED CONSENT & CONSENT TRACKING
    - 2.11.1 Ability to create attributes for the various consent permissions and restrictions which are linked to a specimen or group of specimens (e.g., for a given protocol or site/subset within a protocol) or participant. Accommodate tiered consents.
    - 2.11.2 Ability to store multiple scanned, signed consent forms and have the participant and associated specimens linked to the forms.
    - 2.11.3 Ability to record the date that the consent form was signed by the participant and the name of the person who consented the patient.
    - 2.11.4 Ability to upload a scanned copy of IRB approved (unsigned) consent form(s) and associate them with a project, including the dates of the versions of the consent form.
    - 2.11.5 Ability to capture an electronic signature related to consent form from a password validated user.
    - 2.11.6: Ability to create rules to identify specimens where consent is either not documented or in force when a specimen was taken.
    - 2.11.7 Ability to create Consent workflows specific to a project or protocol for documenting consent.
- 3 IRB PROTOCOL MANAGEMENT (Represents an approved protocol with the IRB)
  - 3.1 Ability to extract protocol specific information from an electronic IRB system.
  - 3.2 Ability to maintain a history of IRB Protocols under which a specimen has been governed.
  - 3.3 Ability to define security model for PHI (Protected Health Information) data access.
- 4 REGULATORY COMPLIANCES
  - 4.1 SYSTEM MEETS REGULATORY COMPLIANCE
    - 4.1.1 System uses appropriate standards that allow TPC/UI Biobank to meet GLP & GMP requirements.
    - 4.1.1.1 System supports secure logging.
    - 4.1.1.2 System supports auditing.
    - 4.1.1.3 System supports archive retention for projects.
    - 4.1.1.4 System enforces accountability.
    - 4.1.1.5 System is implemented to enable non-repudiation.
    - 4.1.2 System uses appropriate privacy standards that allow TPC/UI Biobank to meet HIPAA requirements.
    - 4.1.3 System uses appropriate electronic signature/electronic record standards that allow TPC/UI Biobank to meet 21 CFR Part 11 requirements.
    - 4.1.3.1 Ability to incorporate current and future CAP accreditation standards and ISBER guidelines/standards.
    - 4.1.4 Ability to maintain a complete audit trail, to include at a minimum user ID login, date/times views, queries, edits and log out.
    - 4.1.4.1 Ability to maintain a log of all views of Protected Health Information (PHI) and ability to generate reports on who has seen the PHI for any given participant.
    - 4.1.5 Ability to track grant requirements to share tissue specimens with specific government organizations and/or institutions when specimens were collected under a grant.
  - 4.2 DATA PROVENANCE
    - 4.2.1 Ability to track and report when user changes data.
    - 4.2.2 Ability for protocol version control to track groups of changes over time.
    - 4.2.3 System supports data masking, de-identification, and anonymization workflows in compliance with Federal regulations.
    - 4.2.3.1 System provides tools to remove PHI from textual notes and comments.
    - 4.2.3.2 Ability to store concepts (both PHI and non-PHI) identified from natural-language processing or other de-identification tool in a structured manner (annotation)
    - 4.2.3.3 Ability to generate random IDs and maintain correct relationships between multiple IDs and the specimen.
    - 4.2.3.4 Provides a process to anonymize specimens and specimen attributes for processing and shipment.
- 5 PARTICIPANT ATTRIBUTES
  - 5.1 Ability to specify TPC/UI Biobank (institutional) defined attributes to participant.
  - 5.2 Ability to specify project or individual biobank defined attributes to participant.
  - 5.3 Ability to support variable number of alternate IDs among participants.
  - 5.4 Ability to interface with clinical systems via HL7 v2.x interface.
    - 5.4.1 Ability to batch import participant attributes from daily exports from EHR reporting.
  - 5.5 Ability to track clinic appointments and surgery schedules related to a participant.
  - 5.6 Ability to associate the participant with clinical data contained in other application(s)
  - 5.7 Ability to associate with protocols for enrollment and IRB data.
  - 5.8 Ability to determine data element requirements at protocol level.
  - 5.9 Ability to display information at the participant level.
  - 5.10 Ability to display custom data elements on a participant.
  - 5.11 Ability to notify or prompt for participant's scheduled appointments.
  - 5.12 Ability to register a participant to one or more IRB approved collection protocols and enter information prior to specimen collection.
  - 5.13 Ability to capture and store event or encounter information / data for each specimen.
  - 5.14 Ability to link specimens and participants back to the event or encounter information.
  - 5.15 Ability to store/track/search genotypic and phenotypic data.
    - 5.15.1 Ability to interface with Progeny via API
- 6 ALLOCATING SPECIMENS/SHARING SPECIMENS
  - 6.1 Ability for specimen owner or governing body to request allocation of a specimen or set of specimens.
  - 6.2 Ability for specimens to be designated at the project level by the specimen owner or governing body, as available for sharing (or not)
  - 6.3 Ability to provide a user interface that maps to the federated biorepositories allowing researchers to 'browse' for specimens that may match their research criteria.
- 7 REPORTING AND DATA QUERY TOOL
  - 7.1 GENERAL REPORTING
    - 7.1.1 Ability to run aggregate queries or reports.
    - 7.1.2 Ability to run built-in queries and/or reports.
    - 7.1.3 Ability to create, run and save custom queries and reports at all levels.
    - 7.1.4 System provides a user-friendly graphical user interface to construct custom queries and not command-line SQL.
    - 7.1.5 Ability to create, run and save custom SQL queries.
    - 7.1.5.1 Ability to query across any data field and key system fields.
    - 7.1.5.2 Ability to report the number of times a specimen has been used.
    - 7.1.5.3 Ability to track freezer environmental conditions to enable reporting and alerts.
    - 7.1.5.4 Annotate specimens in batch to reflect adverse events (e.g., freezer wide event)
    - 7.1.6 Ability for the TPC/UI Biobank and each individual biobank to define the basic format and header/footer on each report.
    - 7.1.7 Ability to generate/display report output, printouts, and export results in several different formats.
    - 7.1.8 Provides option to select specific pages of a report to print.
    - 7.1.9 Provides option to specify number of copies of a report.
    - 7.1.10 Ability to run reports based on schedule or set of rules defined at TPC/UI Biobank and individual biobank levels.
    - 7.1.11 Provides report distribution facilities.
    - 7.1.12 Ability to report detailed specimen history including complete chain of custody.
    - 7.1.13 Provides capabilities to cut, paste or import lists of specimens to enable specific reporting.
    - 7.1.14 Ability to plot, graph or display query or report results into meaningful representations.
    - 7.1.15 Ability to organize or group custom reports in a logical fashion.
  - 7.2 USER DEFINED QUERIES
    - 7.2.1 Ability to develop user-defined queries, executed within application's user interface.
    - 7.2.2 Supports queries defined by users with appropriate application permissions.
    - 7.2.3 Ability to save queries for future use.
    - 7.2.4 Ability to share queries with other users and individual biobanks.
    - 7.2.5 Ability to search (with appropriate authorization) for specimens and participants based on existing attributes.
    - 7.2.5.1 Ability to search across protocols and all repositories based on security access.
    - 7.2.6 System provides a separate web-based query tool accessed by external users to generate queries.
  - 7.3 Ability to import data query output into multiple system tables at once, allowing user to create data entry forms 'on the fly'.
  - 7.4 System uses fine-grained security around querying and displaying sensitive information.
  - 7.5 Ability to display query results in user interface table or list.
  - 7.6 Ability to apply restrictions and/or permissions for use based on certain attributes (specific protocol, reason for collection, etc.)
- 8 WORKFLOW SUPPORT
  - 8.1 Ability for the biobank administrator to design a Workflow representing a business rule or series of business rules.
    - 8.1.1 Ability for Workflows to be created for all entities within the biobank.
    - 8.1.2 Ability for Workflows to be built using a Graphical User Interface consisting of pre-defined Workflow steps, which represent actions to be performed.
    - 8.1.3 Ability for Workflows to be built programmatically using an API (application programming interface) provided through some common programming language.
    - 8.1.4 Ability for Workflow steps to include conditional and branching logic, such as IF-THEN-ELSE or CASE steps.
    - 8.1.5 Ability for Workflow steps to include system-related capabilities, such as printing reports, recording audits, sending email, etc.
    - 8.1.5.1 "Ability to send out an HL7 message when an event happens.”
    - 8.1.6 Ability for Workflows to be created in a test environment, and then migrated to the production environment.
    - 8.1.7 Ability for Workflows to be modified after being copied to the production environment without the need to create a version.
    - 8.1.8 Ability for Workflows to be copied or used as templates to build new Workflows.
    - 8.1.9 Ability for Workflows to be versioned.
    - 8.1.10 Ability for Workflows to be run on/applied to biobank entities to cause the Workflow steps to be executed.
    - 8.1.11 Ability for Workflows to be applied to a biobank entity for future execution via a scheduler.
    - 8.1.12 Ability for long-running Workflows to be applied to a biobank entity in a background environment.
    - 8.1.13 Ability to have workflow make a feedback loop (re-doing a past step) until a certain condition is met.
  - 8.2 System supports template features (administrator/software designated and user defined) for biobanking workflows.
    - 8.2.1 Ability for each biobank administrator to define certain rules (required/not required) to be used in workflow templates.
    - 8.2.2 Ability to create custom templates based on a project or workflow.
    - 8.2.3 Ability for a project or biobank to keep data private, with the option to publish (entirely or a subset) to a defined community when workflow is completed or ready for publishing.
    - 8.2.3.1 Ability to provide status of workflow progress (in progress, unpublished, etc.)
  - 8.3 Authorized users can override automatic or scheduled system processes.
- 9 BILLING FUNCTIONS
  - 9.1 Ability to track requested and completed services and determine which are billable.
  - 9.2 Ability to track the services performed and the date the services were performed.
  - 9.3 Ability to compute cost for the use of specimens & shipping based on owning group and characteristics of the specimen.
- 10 TERMINOLOGY MANAGEMENT
  - 10.1 Ability for TPC/UI Biobank to determine and define terminologies or ontologies used within the application.
    - 10.1.1 Ability for the system to pull terminology from an external source `
    - 10.1.2 Ability to use one or more than one terminology standard.
    - 10.1.3 Ability for TPC/UI Biobank administrators to edit, delete, and manage lists of accepted values within the application.
    - 10.1.4 Ability for users to enter ad hoc values for controlled terminology which are flagged for examination later by TPC/UI Biobank administrators.
    - 10.1.5 Ability for system to take values entered in non-standard units and programmatically convert them into the standard units at the time they are entered.
  - 10.2 Ability to map terminologies and ontologies when importing data from other biobanking systems (to maintain interoperability)
    - 10.2.1 Ability to collect and enter data using one terminology/units and have mapping rules to transform the attribute into the standard terminology.
  - 10.3 Ability to control & check annotation based on standard vocabularies for bulk report of specimen annotations.
  - 10.4 System has a mechanism to record and support original and changed annotations with reason for change (e.g., audit trail or support processes for changes to annotation)
  - 10.5 System supports industry terminology standards.
    - 10.5.1 Supports Tissue "Site of Finding” and “Site of Origin” terminology.
    - 10.5.2 System supports preservation of protocol specific terminology, which can be mapped back to standard terminology, to facilitate query functions and interoperability across biobanks and systems.
    - 10.5.3 Ability to capture data elements specific to the protocol.
- 11 SYSTEM FEATURES
  - 11.1 PLATFORM
    - 11.1.1 Ability for the system to be composed of commercial off-the-shelf computer hardware. i.e., not required to be purchased through vendor.
    - 11.1.2 Ability to run the software on more than one type of server.
    - 11.1.3 Ability to scale by adding additional servers when demand increases.
  - 11.2 ARCHITECTURE
    - 11.2.1 Ability for system to accept commands through a public API.
    - 11.2.2 Ability for system to perform data transfers or materialized views via a public API.
    - 11.2.3 Ability to pull or push data on a periodic schedule.
    - 11.2.4 Ability to store information (text fields, labels, etc.) in languages other than US English
    - 11.2.5 Ability to use a replication server to run long queries or reports.
  - 11.3 DATA TRANSFER
    - 11.3.1 Ability to bulk-transfer data out of system on projects, participants, specimens.
    - 11.3.2 Ability to bulk-transfer data into systems on projects, participants, specimens.
    - 11.3.3 Ability to support numerous data transfer formats.
    - 11.3.4 Ability to import data from other biobanking systems to act as a central data aggregation point for a federated system. Migration of data from legacy systems at multiple time points as biobanks join the system.
    - 11.3.4.1 Ability to exchange data on only changed records.
    - 11.3.5 Ability to associate analytic data with a specimen.
    - 11.3.5.1 Ability to support custom analytic data fields.
    - 11.3.6 Ability to associate references to where the data exists after physical analysis of the specimen.
    - 11.3.6.1 Ability to import or attach data files to a specimen.
  - 11.4 USER INTERFACE & CLIENT
    - 11.4.1 Ability to customize the user interface by project at the biobank administrator level.
    - 11.4.2 Ability for each biobank or project to customize their interface without changing the functionality.
    - 11.4.3 System provides online help pages.
    - 11.4.3.1 System provides ability for biobank or project specific help pages.
    - 11.4.3.2 System provides context-sensitive help buttons.
    - 11.4.4 Ability for barcode scanners to auto-advance to next field where appropriate
    - 11.4.5 Ability to for the user interface to be in a language other than US English (internationalization)
    - 11.4.6 Ability for users to connect to the system using any available medium.
    - 11.4.7 Ability for biobanking application to be used on computer running various operating systems.
    - 11.4.8 Ability to use client software on workstations where the use of elevated rights (beyond "user") to install software is not allowed.
    - 11.4.9 Ability for all user functions to be accessible from outside of the Univ of Iowa local area network securely for those authenticated with the proper credentials.
    - 11.4.10 Ability to support users in different time zones.
    - 11.4.11 Ability to access system via web, supports multiple browsers.
    - 11.4.12 Ability to expand capacity and licensing over time to accommodate a growing user base.
  - 11.5 CONFIGURABILITY
    - 11.5.1 Ability for the TPC/UI Biobank developers to configure and associate defined arbitrary attributes to specimen, containers, participants, protocols etc.
    - 11.5.2 Ability for the TPC/UI Biobank to specify required or optional attributes for each specimen type, container type, participant, protocols, etc.
    - 11.5.3 Capacity for rapid real time adjustments to support 11.5.1 and 11.5.2
  - 11.6 SECURITY
    - 11.6.1 Ability to support role-based authorization policies.
    - 11.6.1.1 Ability to create roles with pre-defined sets of permissions.
    - 11.6.1.2 Ability to support permissions by column (i.e., type of data)
    - 11.6.1.3 Ability to support permissions by row (i.e., whose data)
    - 11.6.1.4 Ability to support named user groups to which permissions and rights can be granted or denied.
    - 11.6.1.5 Ability to grant specific security rights at individual, group, project, and biobank levels.
    - 11.6.1.6 Ability to deny specific security rights at individual, group, project, and biobank levels.
    - 11.6.2 Ability to track and manage users by accounts.
    - 11.6.2.1 Ability to integrate with a 3rd party authentication and authorization system.
    - 11.6.2.2 If the proposed solution has separate components (consent management system, specimen management system), then user logs in only once and system maintains credentials for secured access to all system components during the same session without user having to reenter credentials to access each component.
    - 11.6.2.3 Ability to create accounts for users not in the central authentication system.
    - 11.6.2.3.1 Ability to apply TPC/UI Biobank rules and restrictions on usernames and passwords.
    - 11.6.2.3.2 Ability for users to change their password.
    - 11.6.2.3.3 Ability to provide central control of passwords.
    - 11.6.2.3.4 Ability to make passwords expire after a pre-determined number of days (180 days) is the current rule.
    - 11.6.2.4 Ability to lock out users and groups from projects and projects.
    - 11.6.2.5 System keeps passwords secure.
    - 11.6.3 System restricts add, change, query and delete by security level.
    - 11.6.4 Ability to grant or deny access to application functionality by privileged user.
    - 11.6.5 Ability to grant or deny access to application functionality by group role.
    - 11.6.6 Ability to grant or deny access to stored files by user.
    - 11.6.7 Ability to grant or deny access to stored files by group.
    - 11.6.8 Ability to grant or deny access rights to attributes by user.
    - 11.6.9 Ability to grant or deny access to attributes by group.
    - 11.6.10 Ability to alert administrators when multiple security violations exceed a set threshold.
    - 11.6.11 Ability to log-out a user after a TPC/UI Biobank defined inactivity timeout with no delay to re-login.
    - 11.6.11.1 System can restore a user session upon either system failure or system timeout following successful re-authentication by the user.
    - 11.6.12 System provides audit trails automatically provided for all transaction detail.
    - 11.6.13 Ability to track all system configuration changes performed by an administrator. Also known as configuration management or versioning
    - 11.6.13.1 Ability to view reports on all activities for a user and for a group.
    - 11.6.13.2 Ability to view reports on all activity for a specimen.
    - 11.6.14 Upon each change, the system logs field values before and after, date and time of change, identifier of the person who made the change and the host from which change was enabled.
    - 11.6.15 System can de-identify PHI (Protected Health Information) data.
    - 11.6.16 Ability to verify that requestor of specimens has proper credentials.
    - 11.6.17 Data are not transmitted in plain text.
  - 11.7 AVAILABILITY/RECOVERY
    - 11.7.1 Ability to apply upgrades or hot fixes on a live system.
    - 11.7.2 Ability to test upgrades or hot fixes on a test system and migrate the changes to the production system.
    - 11.7.3 Ability to recover after a system failure and not lose data by applying all changes since the last backup to the recovered version.
    - 11.7.4 Ability for entire application hosted by vendor in an emergency.
    - 11.7.5 System provides performance and integrity checks to support monitoring.
    - 11.7.6 System provides critical performance notices to administrator via email.
    - 11.7.7 Ability for administrator to perform system problem resolution including de-bugging.
  - 11.8 API / INTEROPERABILITY
    - 11.8.1 Supports interface to Participant recruitment systems.
    - 11.8.2 Supports interface to Clinical care systems.
    - 11.8.3 Supports interface to Clinical data warehouse.
    - 11.8.4 Supports interface to Pathology systems (list interfaces)
    - 11.8.5 Supports interface to Shipping or courier systems.
    - 11.8.6 Supports interface to National systems to share biospecimens.
    - 11.8.7 Supports interface to External Biobanks
    - 11.8.8 Supports interface to Electronic Medical Records (list existing systems)
    - 11.8.9 Provides rich API or service layer to transfer query data to external software or systems.
  - 11.9 QUALITY ASSURANCE
    - 11.9.1 Vendor supplies a validation plan.
    - 11.9.2 Vendor supplies User Requirements Specifications
    - 11.9.3 Vendor supplies Functional Requirements Specifications
    - 11.9.4 Vendor supplies System Test Scripts
    - 11.9.5 Vendor supplies Installation Qualification Scripts
  - 11.10 VALIDATION
    - 11.10.1 Vendor offers a validation package consisting of a collection of procedural templates, validation test protocols, forms, and scripts designed to help their customers complete the requirements for validation of their product to GLP/GMP regulation levels.
    - 11.10.2 Vendor offers a validation package consisting of a collection of procedural templates, validation test protocols, forms, and scripts designed to help their customers complete the requirements for validation of their product to HIPAA regulation levels.
    - 11.10.3 Vendor offers a validation package consisting of a collection of procedural templates, validation test protocols, forms, and scripts designed to help their customers complete the requirements for validation of their product to 21 CFR Part 11 regulation levels.
    - 11.10.4 Vendor offers validation consultation services such as execution of validation test protocols, either as part of the licensing/maintenance agreement or as an elective option.
- 12 PROJECT FEATURES - Researcher driven.
  - 12.1 ABILITY TO CREATE AND TRACK RESEARCHER PROJECTS AND REQUESTS
  - 12.1.1 Researcher can create, modify, cancel a project.
  - 12.1.2 Researcher can add, modify, cancel specimen requests for a project.
  - 12.1.3 Researcher can search for specimens meeting project requirements.
  - 12.1.4 Researcher can send and receive notices regarding projects specific to the researcher, researcher can receive email or HL7 notices of workflow events related to projects.
  - 12.1.5 Ability to track project approvals across multiple protocols/repositories.
  - 12.1.6 Specimens (or aliquots and derivatives) can be tracked and linked to projects.
  - 12.1.7 Services provided can be tracked and linked to projects.
  - 12.1.8 Specimens can be reserved for a project.
  - 12.1.9 Distributions can be tracked and linked to projects.
  - 12.1.10 Billing can be triggered by distributions (or other workflow events) to projects.
